# Fecal metagenomics for the simultaneous assessment of diet, parasites, and population genetics of an understudied primate

**DOI:** 10.1186/s12983-016-0150-4

**Published:** 2016-04-21

**Authors:** Amrita Srivathsan, Andie Ang, Alfried P. Vogler, Rudolf Meier

**Affiliations:** Department of Biological Sciences, National University of Singapore, 14 Science Drive 4, Singapore, 117543 Singapore; Department of Life Sciences, Imperial College London, Silwood Park Campus, Ascot, SL5 7PY UK; Department of Life Sciences, Natural History Museum, Cromwell Road, London, SW7 5BD UK; Department of Anthropology, University of Colorado Boulder, Boulder, CO 80302 USA; Lee Kong Chian Natural History Museum, 2 Conservatory Drive, Singapore, 117377 Singapore

**Keywords:** Colobines, Banded leaf monkeys, Diet analyses, Metagenomics, Metabarcoding

## Abstract

**Background:**

Rapid habitat loss and degradation are responsible for population decline in a growing number of species. Understanding the natural history of these species is important for designing conservation strategies, such as habitat enhancements or ex-situ conservation. The acquisition of observational data may be difficult for rare and declining species, but metagenomics and metabarcoding can provide novel kinds of information. Here we use these methods for analysing fecal samples from an endangered population of a colobine primate, the banded leaf monkey (*Presbytis femoralis*).

**Results:**

We conducted metagenomics via shotgun sequencing on six fecal samples obtained from a remnant population of *P. femoralis* in a species-rich rainforest patch in Singapore. Shotgun sequencing and identification against a plant barcode reference database reveals a broad dietary profile consisting of at least 53 plant species from 33 families. The diet includes exotic plant species and is broadly consistent with > 2 years of observational data. Metagenomics identified 15 of the 24 plant genera for which there is observational data, but also revealed at least 36 additional species. DNA traces for the diet species were recovered and identifiable in the feces despite long digestion times and a large number of potential food plants within the rainforest habitat (>700 species). We also demonstrate that metagenomics provides greater taxonomic resolution of food plant species by utilizing multiple genetic markers as compared to single-marker metabarcoding. In addition, full mitochondrial genomes of *P. femoralis* individuals were reconstructed from fecal metagenomic shotgun reads, showing very low levels of genetic diversity in the focal population, and the presence of gut parasites could also be confirmed. Metagenomics thus allows for the simultaneous assessment of diet, population genetics and gut parasites based on fecal samples.

**Conclusions:**

Our study demonstrates that metagenomic shotgun sequencing of fecal samples can be successfully used to rapidly obtain natural history data for understudied species with a complex diet. We predict that metagenomics will become a routinely used tool in conservation biology once the cost per sample reduces to ~100 USD within the next few years.

**Electronic supplementary material:**

The online version of this article (doi:10.1186/s12983-016-0150-4) contains supplementary material, which is available to authorized users.

## Background

Human impacts on the environment are responsible for a dramatic increase in habitat destruction and an ever increasing list of species that are in decline. For example, most species of mammals have lost more than half of their original range since the 19^th^ century [1] and for well-studied mammals such as primates over half of the species are listed as endangered [2]. Moreover, rapid habitat loss is responsible for extinction rates that have been estimated to be over 100 times higher than the background rates [3]. The decline of some species can be slowed through conservation measures such as habitat preservation, enhancement or ex-situ management, but such measures require natural history data on the fundamental aspects of the species’ biology, distribution, and genetic diversity. The need for such information is urgent [4], but coincides with the decline of natural history research [5, 6]. Furthermore, the limited resources available are disproportionally spent on a few charismatic species thus leaving little funding for other species [7, 8]. Yet these are likely to represent the majority of the endangered species and populations [9]. There is thus a need for developing new techniques capable of rapidly expanding the data that are obtained in limited field studies often applied to such species.

Valuable natural history information can be obtained by the in-depth study of non-invasive samples such as feces, even if available in small numbers only. Fecal samples are often collected opportunistically during routine field work or they can be obtained efficiently using detection dogs [10]. Such samples have the potential to simultaneously provide information on host genetics, diet, and intestinal parasites. Some of this information can be obtained by direct morphological examination of fecal samples, e.g. by studying diet remnants [11] and gut parasites [12]. Molecular methods have expanded the utility of fecal samples by allowing the analysis of host genetics [13], diet from various sources [14] and the detection of parasites [15]. However molecular methods have been labour intensive as the characterization of multiple species from complex samples involved cloning and Sanger sequencing [14]. The advent of high-throughput sequencing (HTS) has simplified the characterization of complex fecal DNA and now allows for simultaneous characterization of the different aspects of ecology of a species [16]. For fecal samples, HTS can be employed in two ways, either by direct shotgun sequencing of DNA extracted from the fecal samples (metagenomics) or by PCR-based metabarcoding of target genes.

Currently, metabarcoding is more widely used [17, 18], and it has an advantage of lower cost where large numbers of samples have to be screened. Metagenomic shotgun sequencing, in contrast, remains largely unexplored for use in conservation biology [16, 19]. This is presumably due to the higher cost of sequencing and the greater bioinformatics effort required for analysing metagenomic data. But this approach has the potential advantage of rapidly yielding data on genetics, diet, parasites and microbiota from fecal samples, while also avoiding the need for a priori selection of amplification targets which limits the study to the sequencing of a specific subset of the genetic material [20]. This makes metagenomics attractive, but also raises practical challenges. Firstly, bioinformatic challenges arise from the need for a comprehensive reference database against which shotgun data can be queried [16]. Secondly, because of its costs shotgun sequencing is mostly suitable for studies requiring few samples, although with the expectation of cheaper DNA sequencing, one could argue that now is a good time to evaluate and develop the bioinformatic tools for metagenomic data.

The critically endangered population of banded leaf monkeys (*Presbytis femoralis femoralis*) in Singapore is one case where field observational data has been particularly difficult to obtain. Initially described from Singapore and common in the 19^th^ century, the only remaining population now comprises ~40 individuals that are restricted to the Central Catchment Nature Reserve [21]. The forest is surrounded by urban areas and affected by further urban development, which creates conservation challenges including habitat loss, fragmentation and direct anthropogenic disturbance. The situation is exacerbated by the low genetic diversity within the population [22]. Studies of the species’ autecology, prior to developing conservation strategies, have been hampered by the difficulty of making direct observations; a 6-month study in the 1990s led to only 13 sightings [23]. Overall, our current understanding of the species biology is preliminary and here fecal samples can be useful in complementing the current research.

In this study we aim to characterize fecal samples of *P. femoralis* using metagenomics and metabarcoding, for comparisons with field observational data on feeding ecology. Our recent pilot study comparing these approaches for diet analyses in the red-shanked doucs *(Pygathrix nemaeus)* [16] in a controlled zoo environment suggested that shotgun sequencing yields better taxonomic resolution if utilizing multiple reference loci, as compared to single marker metabarcoding, but this was at the expense of lower detection probability of rare food plants in the sample. Here we increased the depth of shotgun sequencing to obtain high taxonomic resolution whilst also detecting rare diet items. We also test whether DNA based analyses are congruent with field observational data, given that this is the first study applying metagenomics to samples collected in the wild. The challenges are considerable because colobine primates have long digestion times that may cause high DNA degradation (Mean Retention Time >40 h [24]), plant barcodes are short and often not species-specific [25], the potential diet of banded leaf monkeys consists of >700 species of trees and lianas in the studied habitat [26], and the amount of target DNA is minute in comparison to the DNA of microbial origin in fecal material. Despite these difficulties, we show that fecal samples can yield a credible set of well-identified plant sequences that correlates with field observational data. In addition, shotgun sequencing provides data on population genetic structure and gut parasites of individual monkeys.

## Results

### Field observations

Two and a half years of field observations yielded 31 feeding observations and banded leaf monkeys were seen to feed on 27 plant species from 24 genera and 20 families during the surveys (Additional file 1: Table S2, Table 1). Diet was primarily comprised of fruits and leaves, and to a lesser degree of flowers. Of the 27 species, *Fibraurea tinctoria*, *Xanthophyllum ellipticum*, *Prunus polystachya* and *Hevea brasiliensis* had two feeding observations each, while feeding on all other species was observed only on a single occasion (Additional file 1: Table S2).

**Table 1:** Summary of plant identifications

Family			Genus			Species		
Annonaceae	GB	●●●	*Drepananthus*	G	●●●	*D. ramuliflorus*	G	●
			*Goniothalamus*	G	●	N/A		
			*Artabotrys*	B		N/A		
Apocynaceae	GB	●●●●	*Hoya*	G	●	N/A		
			*Willughbeia*	G	●●	N/A		
Araceae	GB	●	N/A			N/A		
Bignoniaceae	GB	●	*Radermachera*	GB	●	*R. pinnata*	GB	●
Celastraceae	FGB	●●●	*Salacia*	G	●	N/A		
			*Lophopetalum*	F		*L. multinervium*	F	
Connaraceae	FGB	●●●	*Agelaea*	FG	●●	*A. macrophylla*	F	
Convolvulaceae	FGB	●●●	*Erycibe*	FGB	●●●	*E. tomentosa*	FG	●
Dilleniaceae	FGB	●	*Tetracera*	FGB	●	*T. indica*	FG	●
Erythropalaceae	GB	●●●	*Erythropalum*	GB	●●●	*E. scandens*	GB	●●●
Euphorbiaceae	FGB	●●●●	*Hevea*	FG	●●●●	*H. brasiliensis*	F	
			*Macaranga*	G	●	N/A		
Fabaceae	FGB	●●●●●●	*Bauhinia*	FGB	●●●●	*B. semibifida*	FGB	●●●
			*Dalbergia*	GB	●●●●	*D. parviflora*	G	●
			*Dialium*	G	●	N/A		
			*Entada*	G	●	N/A		
			*Pithecellobium*	GB	●●	*P. clypearia*	GB	●●
			*Pterocarpus*	FG	●	*P. indicus*	F	
			*Falcataria*	F		*F. moluccana*	F	
Gentianaceae	F		*Fagraea*	F		*F. fragrans*	F	
Ixonanthaceae	F		*Ixonanthes*	F		*I. reticulata*	F	
Lamiaceae	GB	●	*Premna*	GB	●	N/A		
Lauraceae	FGB	●●●●●	*Litsea*	FG	●●●●	*L. grandis*	G	●●●
						*L. firma*	F	
						*L. castanea*	F	
						*L. elliptica*	F	
			*Nothaphoebe*	F		*N. umbelliflora*	F	
Loganiaceae	GB	●●●●	*Strychnos*	GB	●●●●	N/A		
Magnoliaceae	G	●	*Magnolia*	G	●	N/A		
Malpighiaceae	GB	●	*Aspidopterys*	G	●	N/A		
Malvaceae	GB	●	*Sterculia*	GB	●	*S. lanceolata*	G	●
Melastomataceae	GB	●	*Pternandra*	GB	●	*P. echinata*	B	
Menispermaceae	FGB	●●●●●●	*Fibraurea*	FG	●●●●●●	*F. tinctoria*	FG	●●●●●●
			*Tinomiscium*	GB	●●	*T. petiolare*	GB	●
			*Tinospora*	G	●●	N/A		
Moraceae	FGB	●●●●●●	*Artocarpus*	FG	●●●●	*A. integer*	G	●●
						*A. elasticus*	F	
			*Ficus*	GB	●●●●	*F. sagittata*	B	
Myristicaceae	FGB	●●●●	*Horsfieldia*	GB	●●	*H. punctatifolia*	GB	●●
			*Knema*	FGB	●●●●	*K. malayana*	F	
			*Myristica*	G	●	*M. elliptica*	G	●
Myrtaceae	F		*Syzygium*	F		*S. grande*	F	
Pandanaceae	GB	●	*Freycinetia*	G	●	N/A		
Passifloraceae	FGB	●●●●	*Adenia*	G	●●●	N/A		
			*Passiflora*	FGB	●●●	*P. laurifolia*	FG	●●●
Pentaphylacaceae	FG	●●	*Adinandra*	FG	●	*A. dumosa*	FG	●
Phyllanthaceae	B		N/A			N/A		
Polygalaceae	FGB	●●●●●	*Securidaca*	G	●●●●	*S. phillippinensis*	G	●●●●
			*Xanthophyllum*	FG	●●●●	*X. ellipticum*	FG	●●●●
						*X. eurhynchum*	F	
Primulaceae	GB	●●	N/A			N/A		
Rhamnaceae	G	●	*Ziziphus*	G	●	*Z. calophylla*	G	●
Rhizophoraceae	FGB	●●●	*Carallia*	G	●	*C. brachiata*	G	●
			*Pellacalyx*	FG	●●●	*P. axillaris*	F	
Rosaceae	FGB	●●●●●●	*Prunus*	FGB	●●●●●●	*P. polystachya*	FGB	●●●●●●
Rubiaceae	GB	●●●●●●	*Mussaenda*	GB	●	N/A		
			*Mussaendopsis*	G	●	*M. beccariana*	G	●
			*Paederia*	G	●	N/A		
			*Psydrax*	G	●●	*P. sp.10*	G	●
			*Uncaria*	GB	●	N/A		
Sapotaceae	FGB	●●	*Madhuca*	F		N/A		
			*Palaquium*	F		*P. xanthochymum*	F	
Smilacaceae	GB	●●●	*Smilax*	G	●●	N/A		
Sapindaceae	FGB	●	*Nephelium*	F		*N. lappaceum*	F	
Symplocaceae	B		*Symplocos*	B		N/A		

Codes following the taxon name represent identifications by F: Field observations, G: Metagenomics and B: Metabarcoding. Underlined: congruent identifications made by metagenomics/metabarcoding and field observational studies. Number of dots represent number of samples from which the identifications were made using metagenomics. All potential misidentifications represented in Additional file 1: Figure S1 were excluded

### Illumina sequencing

Illumina sequencing using HiSeq produced ~67 to ~108 million reads while MiSeq produced ~23 to ~29 million reads per end per sample. For metabarcoding 272,103 to 419,407 sequences per sample were generated for the widely-used marker, P6 loop of *trnL.* These sequences were subsequently filtered and subjected to variant calling and diet identification.

### Diet analysis

BLAST searches of HiSeq and MiSeq metagenomic data were conducted against the plant barcode databases comprising of *rbcL, matK* and *trnL-F* sequences from GenBank and newly sequenced data from the Nee Soon Swamp forest. These yielded between 2616 and 6416 sequence reads (0.004–0.008 %) per sample that could be used for taxonomic classification (Additional file 1: Table S4). A large proportion of the shotgun reads could be classified at least to family (87.0–96.2 %) and a substantial proportion had a genus name associated with them (45.0–56.5 %). A smaller fraction of the reads could be identified to species (27.0–39.5 %), i.e., the reads had similarly high matches to multiple species in a genus or family and thus could be identified only to higher taxonomic ranks (Additional file 1: Table S5). For metabarcoding, after applying the different filtering criteria (FC1 [16], variant calling) we retained the following number of unique sequences per sample: 31 (*BLM1*), 40 (*BLM2*), 31 (*BLM3*), 19 (*BLM4*), 61 (*BLM5*) and 46 (*BLM6*). Here, 4.9–15.8 % of the unique sequences produced species level identifications and 13.1–27.5 % were informative to genus-level, while most contained only family-level information (60.7–73.7 %) (Additional file 1: Table S5).

#### Comparison of metagenomics and metabarcoding

**Fig. 1:**
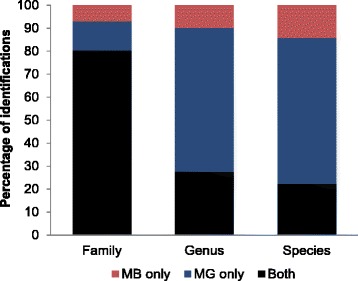
Identifications at different taxonomic hierarchies using metagenomics (MG) and metabarcoding (MB). Colours represent average of proportion of identifications per sample that were made by both MG and MB (*black*), MG only (*blue*), and MB only (*red*)

Family-level identifications were largely congruent between the metagenomic (MG) and metabarcoding (MB) analyses (Fig. 1). Metagenomics yielded identifications for 11–25 families per fecal sample (total number of family-level identifications = 99), while metabarcoding revealed 11–22 families per sample (total number of family-level identifications = 93). The use of 95 % or 90 % identity thresholds led to negligible differences for the metabarcoding results (91 vs. 93 identifications). The performance of the two approaches differed at the genus- and species levels (Fig. 1), as metagenomics generated ~2–3 times more identifications at both taxonomic hierarchies (genus: MG total = 115, range = 11–36 vs MB total = 46, range = 4–11; species: MG total = 59, range 3–21 vs MB total = 24, range = 2–7).

In order to check for the reliability of these identifications, we compared the identified genera/species to the checklists of plants for Nee Soon Swamp forest and Singapore (see Methods). Of the 115 genus-level identifications made by metagenomics, 110 were consistent with the Nee Soon Swamp forest list, while two additional ones matched the Singapore checklist and only three were not known for Singapore (Additional file 1: Figure S1a). The corresponding numbers for metabarcoding were as follows: out of 46 identifications, 40 were for plant genera present in the Nee Soon checklist, one was present only in the Singapore checklist and five identifications were present in neither checklist. Overall both methods were reliable at genus level. At species level, both methods had higher mismatches with the Singapore database, as 13.6 % of metagenomics and 25 % of metabarcoding similarities had best matches to extraneous reference sequences. Note, however, that the comparison between metagenomics and metabarcoding at species level is affected by the small numbers of barcodes corresponding to the P6 loop of *trnL* in the plant database (Additional file 1: Figure S1b).

#### Congruence of DNA based techniques with field observations

We next tested to what degree the pools of diet species inferred by DNA from the six fecal samples overlapped with the field observations. We first excluded potential misidentifications and synonyms (Additional file 1: Figure S1, yellow/red) and then limited the analyses to genus/family level identifications due to greater uncertainty at species level. Using metagenomics we obtained a set of 53 distinct plant identifications from 33 families. Forty-nine of the 53 identifications were at genus level, while four identifications could be made only to family (Araceae sp., Primulaceae sp., Sapidaceae sp., and Sapotaceae sp. Table 1) and could not be resolved further. Using metabarcoding we obtained 35 distinct plant identifications from 32 families. Twenty-one of the 35 identifications were to genus, while the remaining 14 distinct family level identifications could not be resolved further.

**Fig. 2:**
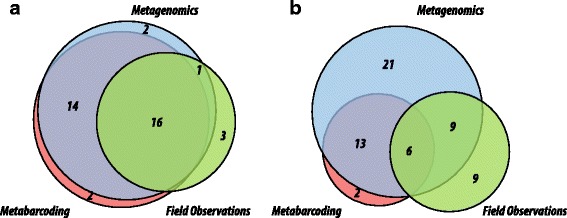
Number of family (**a**) and genus (**b**) level identifications using metagenomics, metabarcoding and field observations

Comparison of these results to diet profile from field studies comprising 27 diet species from 20 families and 24 genera revealed that overall identifications by metagenomics, metabarcoding and field observations corroborated each other, but the DNA based analyses gave larger number of plant identifications. When all three methods were compared, there was high level of congruence for the family level profile with 16 of 20 families of plants from field observations also identified using metagenomics and metabarcoding (Fig. 2a). Due to greater taxonomic resolution achieved by metagenomics, the overlap at genus level was better for metagenomics as compared to metabarcoding (MG: 15/24 genra, MB: 6/24 genera). Lastly, out of the 15 genera observed in HTS based diet analyses and field observations, 11 were found in three or more samples (Table 1). Plants with multiple feeding observations were also present in multiple fecal samples: *Fibraurea, Prunus, Hevea, Xanthophyllum,* and *Litsea* were present in six, six, four, four and four samples respectively.

#### Effect of sequencing depth on diet analysis using metagenomics

**Fig. 3:**
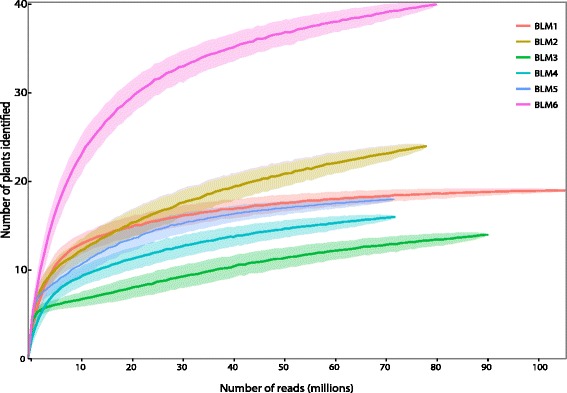
Rarefaction curves representing number of plants identified at varying sequencing depths per sample. Rarefaction of plant reads was extrapolated to estimate effect of rarefaction of all reads in the metagenome

The completeness of the HTS dietary profile may depend on the sequencing depth. Rarefaction of sequence reads indicated that four of six samples approached an asymptote at sequencing depth of 70–100 million reads while the two most diverse samples (*BLM2* and *BLM6*) showed increasing species diversity at this sequencing depth (Fig. 3). Hence, sequencing ~70 million paired reads (~10 Gbp) would lead to identification of most of the diet items in most samples, although due to the variability in diet across individuals or feeding events, the current sequencing depth may not be sufficient to capture the full dietary breadth of an individual.

#### Diet of banded leaf monkeys using metagenomics, metabarcoding and field observational data

We built a dietary profile of *P. femoralis* by combining the above species identifications made from HTS with those from field observations and thus obtained a profile consisting of 38 families. Thirty five of 38 family records could be further resolved to include 60 genera while three family records remained unresolved giving a total of at least 63 plant identifications. We could putatively assign 43 species names to 38 of these genus names (Table 1). They comprise 30 trees, 12 lianas and one shrub. *Fibraurea tinctoria* (Menispermaceae) and *Prunus polystachya* (Rosaceae) were found in all six samples, while *Xanthophyllum ellipticum, Securidaca philippinensis* (Polygalaceae), *Hevea* (Euphorbiaceae), *Bauhinia*, *Dalbergia* (Fabaceae), *Litsea* (Lauraceae), *Strychnos* (Loganiaceae), *Artocarpus*, *Ficus* (Moraceae), *Knema* (Myristicaceae) were present in four samples. The dominant families were Fabaceae, Moraceae, Menispermaceae, Rosaceae and Rubiaceae, which were present across all six samples, and Polygalaceae and Lauraceae present in five samples.

### Low genetic variability in mitochondrial genomes

A complete mitochondrial genome sequence of 16,548 bp was reconstructed for one sample (*BLM5*) and used for read mapping of the remaining samples. The coverage for the six samples was 10.7–104.7X (HiSeq) and 7.4–41.3X (MiSeq). SNP calling using FreeBayes with ploidy = 1 led to identification of only three variables sites in the mitochondrial genomes (Table 2). Four of the six samples showed polymorphism at position 7791 or 15,572 with good confidence of at least 5× coverage for both alleles. Overall, four different genotypes were recognized separating the individuals for *BLM2, BLM4, BLM6* and the three identical samples *BLM1, BLM3* and *BLM5* (Table 2).

**Table 2:** SNP calling for mitochondrial genomes at ploidy =1

Site	*BLM1*	*BLM2*	*BLM3*	*BLM4*	*BLM5*	*BLM6*
7791 (atp8) (T/C)	**10/8**	0/29	**17/22**	2/72	**79/39**	24/3
8155 (atp6) (C/T)	29/0	0/34	17/0	50/0	121/2	15/0
15,572 (*d-*loop) (A/C)	**14/41**	0/30	**6/46**	**8/65**	**88/100**	29/4

SNP calling was based on reads mapped onto reference mitochondrial genome obtained from *BLM5*. Values represent the coverage of two alternative states as shown in first column. Sites are considered polymorphic (highlighted in bold) if both states have at least 5x coverage

### DNA from parasites and other Metazoa in the fecal material

BLAST searches against a parasite SSU rDNA database revealed presence of several protists and nematodes (Table 3). Sequences corresponding to *Blastocystis* and *Entamoeba* were present in varying amounts in four and five samples, respectively*.* Additionally, nematode identifications were made for *Strongyloides* sp. (3 samples) and *Oesophagostomum* sp. (one sample). Using the *COI* database, we recovered sequences mainly corresponding to plants, the primate host and arthropods. Arthropod sequences were found in three samples, and mostly in two (*BLM3, BLM6*; Additional file 1: Table S7), including Muscidae in both samples, and Sarcophagidae and Drosophilidae (*BLM3*) and Sepsidae and Lepidoptera (*BLM6*). At genus level the closest hits were to *Dicranosepsis* (Sepsidae, *BLM6*) and *Phortica* (Drosophilidae, *BLM3*).

**Table 3:** Parasite identifications made using SSU rDNA

Phylum	Order	Genus	*BLM1*	*BLM2*	*BLM3*	*BLM4*	*BLM5*	*BLM6*
(Protozoa)	N/A	*Blastocystis sp.*	271	540	162		1035	
Amoebozoa	Archamoebae	*Entamoeba sp.*	1900	813	725	35		6
Nematoda	Rhabditida	*Strongyloides sp.*	19	11				6
Nematoda	Rhabditida	*Oesophagostomum sp.*						6

The values under the samples represent the number of paired sequences identified. Identifications with counts <5 were excluded

## Discussion

### Comparison of metagenomics, metabarcoding, and field observational data

We demonstrate the power of metagenomic shotgun sequencing for the characterization of fecal samples and find that it can quickly yield important natural history data for endangered species based on few samples. Using metagenomics we document a diverse diet for the banded leaf monkey comprising at least 53 diet plants from 33 families. There was a good overlap between metagenomics and field observational data, with 15 of 24 genera of observed diet plants found in metagenomics data from merely six samples. Moreover, metagenomics recovered similar number of plants as metabarcoding, as suggested by the comparison of family level profiles whilst providing greater taxonomic resolution by using multiple, longer genetic markers. In addition to a very diverse diet, the shotgun approach also detected previously uncharacterized parasites, and revealed low genetic diversity in mitochondrial genomes of *P. femoralis*.

There is good agreement between the diet reconstructed based on HTS data and field observations. Nearly half of the plant genera obtained from observational studies (11/24 genera) were also identified in at least three fecal samples. Researchers are more likely to observe feeding events involving frequently utilized diet species and these are also more likely to be present in multiple fecal samples (e.g., *Fibraurea, Hevea, Prunus*). We thus interpret the good overlap as indirect evidence for the reliability of the diet inferred by metagenomics but note that a dietary profile obtained from six samples is not comprehensive as, e.g., nine of 24 field-observed genera were not detected. Nonetheless, the diet profile obtained with metagenomics was much broader than the profile obtained by field observations. This is not unexpected given that the fieldwork only yielded 31 feeding observations while each fecal sample has the potential to cover ~ 48 h of feeding thus allowing for the identification of rare diet elements. Overall using HTS based methods, our analyses of only six samples added 39 plants to the observational data that had required ~30 months of field work. Field work was still necessary for sample collection, but in the future it can be aided greatly by use of dogs trained to detect feces from target species [10]. However, observational data still has some advantages. Firstly, it can provide information as to which specific individual and which parts of a plant are consumed although the latter can be difficult for food plants in a forest with >700 species of trees and lianas. Secondly, DNA based analyses may not necessarily represent preferred diet plants but also accidental ingestions, such as ingestion of pollen or any other material that may have been associated with the preferred diet items. The latter concern can be overcome by only considering diet items that are found in multiple samples.

When compared to metabarcoding based on one short amplicon, our results are similar to Srivathsan et al. [16] in that the main advantage of metagenomics is higher taxonomic resolution, which can be attributed to utilization of a combination of three barcodes and not limiting the analyses to the P6 loop of *trnL*. Our results for metabarcoding suggest the PCR-based approaches can amplify nearly all plant families revealed by metagenomics if the primers are universal enough, and thus the *trnL* approach is useful when a large number of samples have to be multiplexed and a family-level dietary profile is sufficient. To improve on identifications additional genetic markers can be included using methods such as the two-step approach involving group-specific primers proposed by De Barba et al. [18]. Here, the initial family-level identifications were further resolved using amplicons generated by family/taxon-specific *nrITS* primers. This is feasible, but would require considerable effort for our samples because *trnL* sequences from 18 different families could not be assigned to genus/species suggesting that 18 new primer pairs may need to be designed and then used for each sample. Alternatively, metabarcoding could be based on multiplex PCRs using universal primers for multiple short barcodes. However, there is general consensus in the plant barcoding literature that no specific combination of currently used barcodes can be universally applied for species identification [27] and the multiplex PCRs would have to cover multiple markers. A third option may be anchored hybrid enrichment methods which allow for improving representation of multiple regions of interest in a sample [28]; however additional studies are required to test the feasibility of this approach with degraded DNA from environmental samples.

Metagenomic analysis relies on deep sequencing of DNA extracted and here we used one flowcell of MiSeq in addition to half a lane of HiSeq per sample. In order to explore how much data are needed, we used rarefaction curves to subsample our data. Different samples require different coverage, but overall we find that sequencing ~70 million reads (10 Gbp) per sample would capture the majority of the diet (Fig. 3). Currently, 1 Gbp of data from HiSeq 2500 costs ~40 USD (New York University http://www.med.nyu.edu/ocs/genome-technology-center/services-and-fees) and thus we estimate a cost of ~400 USD per sample. However, new sequencing technologies providing 1 Gbp of data for 7 USD are expected to become available soon, so that the cost will drop to <USD100 [29, 30]. Similarly, while proprietary library preparations can be costly, inexpensive methods for multiplexing are increasingly being developed (e.g. $8–$15 per library [31, 32]). Currently, the cost of metagenomic analysis is nevertheless still substantially higher than the cost of metabarcoding (~25–30 USD for all six samples for the amount of data used here for a single gene), but this comparison excludes manpower expenses which can be high if many genes have to be amplified, multiple primer sets have to be developed, DNA extracts include PCR inhibitors and automated robotic methods are unavailable. Thus the choice of method in future is likely to depend on a multitude of factors including sample size, amount of available data for a species, need for comprehensive characterization, and access to laboratory facilities. The latter is less of a concern for metagenomic studies because the sequencing of extracted DNA can be outsourced.

With coverage of 10 Gbp we were able to reveal nearly all of the plants that were identified using metabarcoding. Our results differ somewhat from the results of Srivathsan et al. [16], where metagenomics could not reveal as many plants as metabarcoding. The difference between the two studies was likely due to the >5-fold larger number of reads corresponding to the plant chloroplast barcode regions in the metagenomes of the banded leaf monkey as compared to the earlier study on *Pygathrix nemaeus*. The reason for the greater amount of plant signal in the current study is unclear. The difference between the studies could be related to physiological differences between captive vs. wild populations or biological differences between the two species that may exist despite their close relationships and similar herbivorous life style. There may also be differences in the types of materials ingested, i.e. young vs. old leaf material or fruits vs. leaves, which may in turn lead to differential rates of digestion and thereby influence the eventual contribution to DNA in fecal samples [33]. These results suggest that the data requirements for metagenomics should be assessed on a species-by-species basis.

### Implications for biology and conservation of the *P. femoralis* in Singapore

We obtained a diverse diet profile for the banded leaf monkey consisting of at least 63 plants from 38 families, with a few plants dominating, such as *Fibraurea tinctoria* and *Prunus polystachya* that were present across all six samples. Our results also suggest that lianas are a major component of the diet (28 % of species identified). Besides these, certain introduced plant species such as *Hevea* (Brazilian rubber tree) are key components of the diet (four samples, and two field observations), which suggests that the removal of non-native plant species may under certain conditions negatively impact primate populations. However, the current data suggest that conserving *P. f. femoralis* does not require the focus on any single plant species, while the great diet breadth highlights the importance of the integrity of the forest and its high species richness. For future conservation efforts, a wide range of diet species identified during this study should be planted to connect forest fragments and thereby facilitate migratory movement of the species. This is particularly relevant given the recent construction of EcoLink which connects two forest fragments in Singapore allowing a corridor for the movement of the primates from its existing habitat (Central Catchment Nature Reserve) to a forest where the primates became extinct in 1980s (Bukit Timah Nature Reserve) [34].

In terms of population genetics, the mitochondrial genomes show very little variability, which is in agreement with the data from a previous study using the *d-loop* alone that found only one variable site across the six samples [16]. With six complete mitochondrial genomes now available, we find only two additional variable sites. In addition, we identified polymorphic sites in four of the six samples. The two most likely explanations for the polymorphisms are: 1) reads from nuclear copies of mitochondrial DNA (NuMTs) were mapped onto the reference genome, and 2) presence of multiple copies of mitochondrial genomes in the organism (heteroplasmy) [35]. Given that the two different bases were present at nearly same ratio in at least one of the samples (Table 2), we consider the latter explanation more likely. This is because regions from nuclear genomes are unlikely to be represented at similar frequency as mitochondrial genomes. For example, *in silico* examination of human genome has shown that at any given position of mtDNA, 1–46 copies of NuMTs can be mapped from one nuclear genome; on the other hand, for every nuclear genome thousands of copies of mitochondrial genomes are likely to be present [36].

In addition to diet and host mitochondrial genomes, we also characterized a number of sequences corresponding to gut parasites. Most of them were from common parasites (*Blastocystis, Entamoeba*, *Strongyloides*). One sample (*BLM6*) deviated from the others by containing sequences putatively identified as *Oesophagostomum*, which has previously been reported in Southeast Asia (Malaysia) [37]. These parasites can have major impact on host health and thus influence population survival and reproduction. *Strongyloides* infections can be potentially fatal in primates [38]. Besides the negative effects on the primate, some of these parasites such as *Blastocystis, Oesophagostomum, Strongyloides* are zoonotic and thus present potential public health issues in an urban environment [39]. The detection of these parasites calls for a closer monitoring of the primate population. For example, with putative identifications for the parasites made here, any additional samples obtained can be screened for the parasites either in a targeted manner using PCR amplification with specific primers or via shotgun sequencing. This will help determine the prevalence of these gut parasites in the population over time.

## Conclusions

Characterizing non-invasively collected fecal samples using metagenomics can greatly complement field based research of understudied animals and help with rapidly generating data where urgent conservation intervention is required. However, there are several future challenges that remain to be addressed. Firstly, more species need to be studied in order to understand the data requirements for metagenomic analyses, which could differ between dietary types such as herbivory, carnivory or omnivory. The results of these studies should be compared with direct feeding observations and results based on metabarcoding. Secondly, in the current study, we used three plant barcodes and a single metazoan barcode to characterize diet and other interactions. With short reads and a small number of barcodes, some false positives are expected and observed, especially at species level resolution (Additional file 1: Figure S1, red). Such false positives should become less common as more mitochondrial and chloroplast reference genomes become available [40]. These developments are also likely to improve the resolution of identifications. As these studies progress, we predict that metagenomics will become a powerful and important tool for studying the ecology of endangered species with different dietary requirements and biology.

## Methods

### Field survey and observational data

Field surveys were conducted between 2008 and 2011 in the Nee Soon Swamp forest and adjacent forests within the Central Catchment Nature Reserve [21]. Once a monkey was detected, it was followed for as long as possible and feeding observations were recorded whenever it manually or orally handled food and brought it to the mouth. At the same time, fecal samples were collected opportunistically [22] whenever defecation was observed. The samples were stored at -70 °C. Note that samples collected were from different days and locations which were separated by man-made barriers (military infrastructure), thus increasing the likelihood that they were from different groups of monkeys [22].

### DNA extraction and High-throughput Sequencing

DNA was extracted from ~150 mg of fecal sample using QIAGEN DNeasy Blood and Tissue Kit as described in Srivathsan et al. [16]. Although QIAGEN stool extraction kit would be an alternative for fecal extractions as it reduces PCR inhibition, extraction of DNA using this kit has previously shown consistent amplification of contaminant potato DNA [41] and hence this was not used. For each extraction, the interior of the feces was randomly sampled. Care was taken to avoid contamination and samples were extracted in a lab where no molecular work on plants had been carried out. The outside layer of the fecal sample was furthermore avoided in order to minimise contamination. DNA extractions from six fecal samples (henceforth called *BLM1*-*6*) were sent for shotgun sequencing using Illumina HiSeq and MiSeq platforms. For HiSeq sequencing, one library was constructed for each fecal sample (fragment size 280–300 bp). Two samples were multiplexed in one lane of Illumina HiSeq 2000 (Illumina Inc., San Diego, CA) and paired 76 bp reads were obtained using TruSeq PE Cluster Kit v3 and TruSeq SBS Kit v3. Additionally, Illumina MiSeq was used to generate paired 300 bp reads. Here the libraries were prepared using TruSeq Nano DNA sample preparation kit, with insert sizes of ~700 bp. Data were generated using one run of the MiSeq per sample.

For the metabarcoding experiment, we used two sets of samples: the first set comprised four samples with the same extractions (*BLM1*, *BLM3*, *BLM4*, *BLM6*) that were used for metagenomics. The second set comprised different extractions from the same samples that were used for metagenomics (*BLM2* and *BLM5*). For all six, three replicates of 45-cycle PCR reactions were carried out for amplifying the P6 loop of chloroplast *trnL* intron using primers *trnL*-g and *trnL*-h [42] in a procedure identical to Srivathsan et al. [16]. The three replicates were pooled and Illumina MiSeq was then used to obtain ~200,000–400,000 paired reads of 150 bp; the libraries were prepared using the TruSeq Nano DNA sample preparation kit (150 PE).

### Databases for identifications

In order to perform a multidimensional characterization of the fecal samples we built several reference databases. First, for diet analyses we generated databases corresponding to three plant barcodes: *matK* (73,891 sequences from 7894 genera and 410 families), *rbcL* (60,989 sequences from 7539 genera, and 421 families) and *trnL-F* (37,747 sequences from 5053 genera and 281 families). In order to obtain these databases, we first included data available from GenBank and processed it to retain only the homologous sets of sequences using a pipeline developed by Hunt et al. [43]; i.e., a curated set of sequences from our lab and GenBank was matched using BLASTN to all downloaded sequences from GenBank for the three barcodes. Using subject start and end position of matches in the BLAST output, homologous regions were retrieved. We also included 186, 224 and 195 newly generated barcodes for *matK, rbcL* and *trnL-F* respectively for plant species from Nee Soon Swamp Forest (Additional file 1). Database for metabarcoding comprised *trnL* P6 loop sequences obtained from the *trnL* database generate for metagenomics using ecoPCR [44], which uses an *in silico* PCR approach, given that most of reference *trnL* sequences contain primer regions that can be used to extract the homologous sections of the sequences. This database contained 31,008 sequences from 4813 genera and 240 families. In order to detect parasite DNA in the samples, a targeted database of common non-human primate parasites was compiled based on a literature survey (Additional file 1: Table S1) and contained 5557 sequences corresponding to SSU rDNA (18S) from 24 genera. Lastly, a COI database comprising 765,218 sequences from Eukaryota was extracted from GenBank using the same pipeline by Hunt et al. [43] as described above. Further details for database generation are provided in Additional file 1.

### Data analysis

Prior to analyses, FASTQ files were trimmed using Trimmomatic v 0.32 [45] to remove adapter sequences and low quality sequences (Average quality score = 30, minimum length = 50) after removal of all bases below average score of 20 at the start and end of sequences (LEADING = 20, TRAILING = 20).

#### Diet

For diet identification using metagenomics, we followed the protocol developed in Srivathsan et al. [16], which determined that identification of plant sequences with accuracy was satisfied if *rbcL, matK* and *trnL-F* reference barcodes share a minimum of 50 bp overlap with a given read at a minimum of 98 % identity, and at least two barcodes produced the same identification at a given hierarchical level. In an initial step MEGABLAST searches (word-size = 28) for each end of the paired-end data were conducted independently against the three plant barcode databases, after which the extracted sequences were filtered for a minimum of 50 bp overlap and 98 % identity threshold. An additional filtering step was used, where all alignments with incomplete overlaps were excluded. *readsidentifier* (v 1.0) [16] was then used to assign each read to the lowest identifiable taxonomic levels. We obtained a species level identification for a read if the best identity BLAST hit was to reference sequence(s) from a single species. We obtained a genus level identification if a read best matched to two or more species from one genus and likewise family level identifications were made based on multiple best hits to two  or more genera of the same family  (similar to the Lowest Common Ancestor algorithm [46]). Next, the results for the two paired-ends were compared, and the pair was retained only if the identifications were not in conflict at a given taxonomic hierarchy (paired-end analyses) (see [16]). We performed this procedure for each of the three barcode genes and recorded whether a given identification (species/genus/family) was made using one, two or three genes. All diet items identified using only one of the barcodes were excluded.

In order to obtain a diet-estimate from amplicon-based metabarcoding, paired-end reads were merged using *illuminapairedend* tool in OBITOOLS [47]. Sequences were assigned to different samples using *ngsfilter* after which unique reads were obtained using *obiuniq*. All sequences ≤10 bp were excluded using *obigrep*. Next, we filtered the data based on sequence counts where sequences with counts <100 were first removed followed by removal of all sequences with counts <0.1 % of the counts of the most dominant signal (FC1, [16]). Variant calling was then performed using *obiclean*, where we identified sets of sequences that differed by only 1 bp from each other. Within such a set, the sequence with the maximum count was labelled “head” while the variants were called “internal”. Sequences that did not have any variants were tagged as “singleton”. Only “head” and “singleton” sequences were used for taxonomic assignment. Identifications to genus/species were then made using *ecotag* and the *trnL* P6 loop database under the threshold 95 % identity. For identifications to family we compared both 95 % [18] and a more relaxed 90 % identity criterion.

The identifications made by metagenomics and metabarcoding were compared against the known flora of the monkeys’ habitat using a checklist of the Nee Soon Swamp forest [26] and the checklist of angiosperms of Singapore [48]. Identifications were considered most reliable if they were to a plant from the Nee Soon Swamp forest checklist, followed by Singapore checklist and least reliable if not known from Singapore.

#### Relationship between sequencing depth and diet diversity

In order to determine how sequencing depth affects the recovery of diet species, we selected for each sample all reads that matched plant barcodes and were used for plant identifications. This set of reads was then rarefied 1000 times. Each rarefied subset was analysed using the same identification criteria that were described previously (98 % identity, 50 bp overlap, minimum two barcodes). We then plotted the number of identified plants against sequencing depth. Here only genus and family level identifications were considered.

#### Mitochondrial genomes

Mitochondrial genomes of *P. f. femoralis* were obtained by metagenomic assembly on one samples (*BLM5*) using MITObim [49] under default parameters and the mitochondrial genome of the related *P. melalophos* (GenBank: NC_008217) as reference. The genome was annotated using MITOS [50] and further manually curated prior to GenBank submission. A single contig was obtained against which reads were mapped back from all six HiSeq and MiSeq datasets using BWA *mem* [51]. The bam files generated in this process were further filtered to retain only sequences with mapping quality of at least 30 and available paired-end reads. We used FreeBayes [52] with ploidy = 1, maximum read mismatch to reference setting at 5 %, minimum coverage for alternate allele at five and variant quality score of at least 30 to identify the variant sites across the six samples. Results obtained using HiSeq and MiSeq datasets from the same sample were first cross checked to ensure there were no differences in the calls for the two runs and then summarised together (see Additional file 1).

#### Parasites and other eukaryotes

In order to characterize other eukaryotes represented in the fecal samples, reads were matched against *COI* databases using settings identical to those in the diet analyses. All reads matching the *COI* database were retrieved and matched to the NT database of GenBank. Identifications were filtered using *readsidentifier* v1.0 at 95 % and 98 % identities, and only complete overlap between a read and *COI* sequences was considered. An initial survey of these results revealed matches to mostly plant, primate and insect sequences. Given that we were also interested in identifying potential parasites, we built a target rDNA database of common non-human parasites (Additional file 1: Table S1). SSU rDNA was selected as it has often been used to barcode single-cellular organisms and parasites such as nematodes. Similar to *COI* analyses, we matched the sequences using MEGABLAST and the retrieved hits were then matched to the NT database to validate the results. The reads were then classified at 98 % similarity and 50 bp overlap using *readsidentifier* v1.0.

## Availability of supporting data

Barcode sequences that matched metagenomics data have been submitted to GenBank with accession numbers KU853075-KU853258. Reference mitochondrial genome has been submitted to GenBank under accession number KU899140. Sequences corresponding to plants from the metagenomic data and the metabarcoding dataset, and plant databases have been archived in LabArchives doi:10.6070/H4000047. COI and parasite databases are available on request. Scripts written specifically for the study are included in the *readsidentifier* package https://github.com/asrivathsan/readsidentifier.

## Additional file

Additional file 1: Supplementary methods, figures and tables. (DOCX 66 kb)

## Abbreviations

HTS: high throughput sequencing
MG: metagenomics
MB: metabarcoding
