# Supplementary material for: Fecal metagenomics for the simultaneous assessment of diet, parasites, and population genetics of an understudied primate
Source: Front Zool. 2016 Apr 21;13:17. doi: 10.1186/s12983-016-0150-4 (PMC4839110; doi:10.1186/s12983-016-0150-4)
Supplement: Additional file 1: — Supplementary methods, figures and tables. (DOCX 66 kb) [file 12983_2016_150_MOESM1_ESM.docx]

**Supplementary Materials**

**Supplementary Methods**

*DNA extraction and sequencing for plant barcoding*

For DNA extraction, tissues were ground using liquid nitrogen and extraction was carried out either with the CTAB method as described by Kutty *et al.,*(2007) or by modified method by Doyle & Doyle (1987) [1-3]. The primer pairs used were as follows (annealing temperatures are in brackets): rbcLa_f and rbcLa_rev (54-55°C) [4], 3F_KIM f and 1R_KIM r (52°C) (Kim Ki-Joong, unpublished) and trnL c and f (52-55°C) [5]. The PCR reactions were done using the following conditions: Initial denaturation at 95°C for 5 min, followed by 35 cycles of 94°C for 1 minute, annealing for 1 min and 72°C for 1 min 30 sec. Final extension was at 72°C for 5 min. Gel extractions were performed if there were multiple bands present after optimization of conditions. The amplified PCR products were purified with SureClean (Bioline, Randolph, MA). Cycle sequencing was performed using BigDye Terminator v3.1 and products were analysed in both directions on an ABI 3100 Genetic Analyser (Perkin Elmer, Waltham, MA). Sequences were edited with Sequencher v 4.6 (Gene Codes Crop, Ann Arbor, MI, USA).

*Building databases from GenBank data*

We generated a database comprising all barcode sequences available at GenBank for *rbcL*, *matK*, and *trnL-F* as of June 2014*.* Here we first used a curated set of sequences containing the barcode region only. The sequences were then matched to the downloaded sequences from GenBank (search limited to “Magnoliophyta”[organism]) using BLASTN, evalue 1e-5. We retrieved a homologous subset of the downloaded sequences by parsing the BLAST outputs using pipeline of Hunt et al. (2007) [6] to obtain a set of sequences retaining only the barcode region from the available GenBank records. All sequences shorter than 50% of the longest sequence in the curated sequence set were discarded. This yielded a set of sequences that were limited to the region of interest. Lastly, we aligned the *rbcL* and *matK* datasets using MAFFT v7 [7] and trimmed the edges to ensure the homology for these genes.

*Validation of mt-genome polymorphisms*

SNP calling using FreeBayes was run for MiSeq and HiSeq data individually. In order to compare SNP calling from the two datasets from the same samples, we used the coverage per allele in each datasets and checked for consistency (Table S6). Once validated, the values were added to yield the total coverage per site. For one of the variant sites we had additional data from Ang et al. (2012) [8], which confirmed the polymorphism. Lastly, we applied the coverage of 5X to ensure reduce the effect of nuclear genome paralogs of the mitochondrial genes in influencing the analyses.

Figure S1: Identifications made by metagenomics and metabarcoding to genus (a) and species (b). Green: plants present in Nee Soon Swamp forest checklist, yellow: plants present in Singapore checklist and red: plants absent in either checklists. Names highlighted in red do not have corresponding *trnL* p6 loop sequence in the database and hence cannot be used for comparisons. BLM1-6 are represented by 1-6.

(a)

­

(b)

**Supplementary Tables**

## Table S1 List of parasitic taxa in our non-human anthropoid primate SSU rDNA database

| Parasite | Host species | Location | Reference |
| --- | --- | --- | --- |
| *Ancyclostoma* | *Erythrocebus patas* | Africa | Adedokun *et al.* (2002) |
| *Ascaris* | *Macaca nigra, Macaca mulatta, Semnopithecus entellus* | Asia | Jones-Engel *et al.* (2004), Remfry (1978), Parmar *et al.* (2012) |
| *Balantidium* | *Chlorocebus aethiops, Cercopithecus mitis, Cercocebus torquatus, Lophocebus aterrimus, Papio cynocephalus, Cercopithecus neglectus, Pan troglodytes, Nomascus leucogenys, Erythrocebus patas* | Africa | Muriuki *et al.*, 1998, Munene *et al.* (1998), Karere and Munene (2002), Adedokun *et al.* (2002) |
| *Bertiella* | *Colobus guereza, Cercopithecus ascanius, Macaca fuscata, Papio ursinus, Trachypithecus cristatus* | Africa,Asia | Chapman *et al.*(2005), Gotoh (2000), Goldsmid (1974), Palmieri *et al.* (1980) |
| *Blastocystis* | *Macaca nigra, Macaca nigrescens, Macaca hecki, Macaca tonkeana, Macaca maura, Macaca ochreata, Macaca fascicularis, Macaca nemestrina, Papio cynocephalus, Chlorocebus aethiops, Lophocebus albigena, Procolobus rufomitratus* | Asia, Africa, Captive | Jones-Engel *et al.* (2004), Legesse *et al.* (2004), Chapman *et al.* (2011), Srivathsan et al. (2015) |
| *Cryptosporidium* | *Chlorocebus aethiops,Papio cynocephalus, Macaca sinica, Semnopithecus priam, Trachypithecus vetulus* | Asia, Africa | Legesse *et al.* (2004), Ekanayake *et al.* (2006) |
| *Dicrocoeliidae* | *Colobus guereza, Cercopithecus ascanius* | Africa, | Chapman *et al.*(2005), |
| *Dipetalonema* | *Saguinus geoffroyi, Aotus trivirgatus, Ateles fusciceps, Ateles geoffroyi, Cebus capucinus, Trachypithecus obscurus* | Asia, Neotropics | Thatcher and Porter (1968), Mak *et al.* (1980) |
| *Endolimax* | *Macaca nigra, Lophocebus albigena, Procolobus rufomitratus, Cercopithecus ascanius, Colobus guereza, Cercopithecus mitis* | Asia, Africa | Jones-Engel *et al.* (2004), Chapman *et al.* (2011) |
| *Entamoeba* | *Piliocolobus tephrosceles, Colobus guereza,Cercopithecus ascanius, Papio cynocephalus, Chlorocebus aethiops, Cercopithecus mitis, Cercocebus torquatus, Lophocebus aterrimus, Macaca nigra, Micaca nigrescens, Macaca hecki, Macaca tonkeana, Macaca maura, Macaca ochreata, Macaca fascicularis, Semnopithecus entellus, Lophocebus albigena, Procolobus rufomitratus, Cercopithecus neglectus, Colobus angolensis, Symphalangus syndactylus, Gorilla gorilla, Pan troglodytes, Hylobates lar* | Africa,Asia, Captive | Chapman *et al.*(2005), Muriuki *et al.*, 1998, Jones-Engel *et al.* (2004), Munene *et al.* (1998), Legesse *et al.* (2004), Parmar *et al.* (2012), Chapman *et al.* (2011), Karere and Munene (2002), Gillespie *et al.* (2005), Levecke *et al.* (2007), Srivathsan et al. (2015) |
| *Enterobius* | *Cercopithecus ascanius, Macaca sinica, Macaca mulatta, Papio cynocephalus, Cercopithecus mitis, Papio ursinus, Trachypithecus cristatus, Erythrocebus patas* | Africa,Asia | Chapman *et al.*(2005),Dewit *et al.* (1991), Remfry (1978) Munene *et al.* (1998), Goldsmid (1974), Palmieri *et al.* (1980), Adedokun *et al.* (2002) |
| *Giardia* | *Cercopithecus ascanius, Procolobus rufomitratus, Lophocebus albigenus, Symphalangus syndactylus, Gorilla gorilla, Hylobates lar, Nomascus leucogenys* | Africa, Captive | Chapman *et al.*(2005), Chapman *et al.* (2011), Levecke *et al.* (2007) |
| *Hymenolepis* | *Semnopithecus entellus, Macaca sinica, Macaca mulatta* | Asia | Dewit *et al.* (1991), Remfry (1978) |
| *Oesophagostomum* | *Procolobus rufomitratus, Colobus guereza,Cercopithecus ascanius, Macaca arctoides Macaca sinica, Macaca mulatta, Cercopithecus mitis, Macaca fuscata, Papio ursinus, Trachypithecus cristatus, Pan paniscus* | Africa, Asia | Chapman *et al.*(2005), Nath *et al.*, 2012, Remfry (1978), Munene *et al.* (1998), Gotoh (2000), Goldsmid (1974), Palmieri *et al.* (1980), Hasegawa *et al.* (1983) |
| *Physaloptera* | *Macaca sinica, Macaca mulatta, Saguinus geoffroyi* | Asia, Neotropics | Dewit *et al.* (1991), Thatcher and Porter (1968) |
| *Schistosoma* | *Papio cynocephalus, Cercopithecus mitis, Papio ursinus* | Africa | Munene *et al.* (1998), Goldsmid (1974) |
| *Spirometra* | *Papio cynocephalus, Semnopithecus entellus, Macaca mulatta, Saguinus geoffroyi* | Africa, Asia, Neoptropics | Nobrega-Lee *et al.* (2007), Parmar *et al.* (2012), Thatcher and Porter (1968) |
| *Streptopharagus* | *Cercopithecus ascanius, Macaca sinica, Macaca mulatta, Papio cynocephalus, Macaca fuscata, Papio ursinus, Cercopithecus neglectus* | Africa,Asia | Chapman *et al.*(2005), Dewit *et al.* (1991), Munene *et al.* (1998), Gotoh (2000), Goldsmid (1974), Karere and Munene (2002) |
| *Strongyloides* | *Procolobus rufomitratus, Colobus guereza,Cercopithecus ascanius, Papio cynocephalus, Chlorocebus aethiops, Cercopithecus mitis, Cercocebus torquatus, Lophocebus aterrimus, Macaca sinica, Macaca mulatta, Macaca fuscata, Papio ursinus, Semnopithecus entellus, Cercopithecus neglectus, Colobus angolensis, Symphalangus syndactylus. Nomascus leucogenys, Pygathrix nemaeus, Erythrocebus patas, Pan paniscus* | Africa,Asia, Captive | Chapman *et al.*(2005), Muriuki *et al.*, 1998, Dewit *et al.* (1991), Remfry (1978), Legesse *et al.* (2004), Gotoh (2000), Goldsmid (1974), Paramar *et al.* (2012), Karere and Munene (2002), Gillespie *et al.* (2005), Levecke *et al.* (2007), Srivathsan et al. (2015), Adedokun *et al.* (2002), Hasegawa *et al.* (1983) |
| *Taenia* | *Chlorocebus aethiops, Erythrocebus patas* | Africa | Sulaiman *et al.* (1986) |
| *Trichostrongylus* | *Macaca sinica, Macaca mulatta, Papio cynocephalus, Cercopithecus mitis, Papio ursinus* | Asia, Africa | Dewit *et al.* (1991), Munene *et al.* (1998), Goldsmid (1974) |
| *Trichuris* | *Procolobus rufomitratus, Colobus guereza,Cercopithecus ascanius, Papio cynocephalus, Chlorocebus aethiops, Cercopithecus mitis, Cercocebus torquatus, Lophocebus aterrimus, Trachypithecus geei, Macaca sinica, Macaca hecki, Macaca tonkeana, Macaca fuscata, Papio ursinus, Semnopithecus entellus, Colobus angolensis, Nomascus concolor, Trachypithecus francoisi, Hoolock hoolock, Erythrocebus patas, Pan paniscus* | Africa,Asia, Captive | Chapman *et al.*(2005), Muriuki *et al.*, 1998, Dewit *et al.* (1991), Jones-Engel *et al.* (2004), Gotoh (2000), Goldsmid (1974), Parmar *et al.* (2012), Gillespie *et al.* (2005), Levecke *et al.* (2007), Liu *et al.* (2013), Nath *et al.* (2012), Adedokun *et al.* (2002), Hasegawa *et al.* (1983) |
| *Trypanosoma* | *Macaca silenus, Saimiri sciureus, Macaca mulatta* | Captive, Asia, Neotropics | Pung *et al.* (1998), Ziccardi and Lorenco-de-Oliveira (1997), Fulton and Harrison (1946) |
| *Trypanoxyuris* | *Alouatta pigra, Aotus trivirgatus, Ateles fusciceps, Ateles geoffroyi, Saguinus geoffroyi,* | Neotropics | Thatcher and Porter (1968) |

Table S2. List of species identified as diet plants for the banded leaf monkeys using observational data

| S.No. | Species | Family | Material fed (leaves/fruits) | Number of observations |
| --- | --- | --- | --- | --- |
| *1* | *Adinandra dumosa* | Pentaphylacaceae | Flowers | 1 |
| *2* | *Agelaea macrophylla* | Connaraceae | Fruits | 1 |
| *3* | *Artocarpus elasticus* | Moraceae | Fruits | 1 |
| *4* | *Bauhinia semibifida* | Fabaceae | Leaves and flowers | 1 |
| *5* | *Erycibe tomentosa* | Convolvulaceae | Leaves | 1 |
| *6* | *Fagraea fragrans* | Gentianaceae | Leaves | 1 |
| *7* | *Falcataria moluccana** | Fabaceae | Leaves | 1 |
| *8* | *Fibraurea tinctoria* | Menispermaceae | Leaves and flowers | 2 |
| *9* | *Hevea brasiliensis* | Euphorbiaceae | Leaves | 2 |
| *10* | *Ixonanthes reticulate* | Ixonanthaceae | Fruits | 1 |
| *11* | *Knema malayana* | Myristicaceae | Fruits | 1 |
| *12* | *Litsea castanea* | Lauraceae | Leaves | 1 |
| *13* | *Litsea elliptica* | Lauraceae | Fruits | 1 |
| *14* | *Litsea firma* | Lauraceae | Fruits | 1 |
| *15* | *Lophopetalum multinervium* | Celastraceae | Fruits | 1 |
| *16* | *Madhuca sp.* | Sapotaceae | Fruits | 1 |
| *17* | *Nephelium lappaceum* | Sapindaceae | Fruits | 1 |
| *18* | *Nothaphoebe umbelliflora** | Lauraceae | Leaves | 1 |
| *19* | *Palaquium xanthochymum* | Sapotaceae | Fruits | 1 |
| *20* | *Passiflora laurifolia* | Passifloraceae | Leaves | 1 |
| *21* | *Pellacalyx axillaris* | Rhizophoraceae | Fruits | 1 |
| *22* | *Prunus polystachya* | Rosaceae | Fruits | 2 |
| *23* | *Pterocarpus indicus* | Fabaceae | Leaves | 1 |
| *24* | *Syzygium grande* | Myrtaceae | Leaves | 1 |
| *25* | *Tetracera indica* | Dilleniaceae | Fruits | 1 |
| *26* | *Xanthophyllum ellipticum* | Polygalaceae | Fruits | 2 |
| *27* | *Xanthophyllum eurhynchum* | Polygalaceae | Leaves | 1 |

Table S3: Number of reads per sample for the metagenomic Illumina HiSeq and Illumina MiSeq datasets and the metabarcoding experiment

| Sample | HiSeq (paired reads) | | MiSeq (paired reads) | | Metabarcoding |
| --- | --- | --- | --- | --- | --- |
|  | Raw data | Post Q30 |  | Post Q30 |  |
| BLM1 | 107,675,433 | 89,771,150 | \| 28,715,570 \| \| --- \| | 16,283,015 | 338,131 |
| BLM2 | 72,660,997 | 58,974,905 | \| 27,760,062 \| \| --- \| | 19,546,740 | 385,907 |
| BLM3 | 85,963,340 | 72,141,213 | 26,595,637 | 18,507,608 | 371,919 |
| BLM4 | 66,986,068 | 55,260,242 | 23,190,419 | 17,062,159 | 272,103 |
| BLM5 | 68,188,666 | 54,961,193 | \| 27,840,572 \| \| --- \| | 17,200,693 | 419,407 |
| BLM6 | 76,440,420 | 63,307,902 | 26,591,829 | 17,208,334 | 320,270 |

Table S4: Number of plant barcode reads from the metagenomics data used for identifications per sample

| **Sample** | **matK** | **rbcL** | **trnL-F** | **Total** |
| --- | --- | --- | --- | --- |
| BLM1 | 2327 | 1030 | 3059 | 6,416 |
| BLM2 | 1800 | 978 | 2324 | 5,102 |
| BLM3 | 1223 | 498 | 1519 | 3,240 |
| BLM4 | 1304 | 793 | 2010 | 4,107 |
| BLM5 | 986 | 537 | 1093 | 2,616 |
| BLM6 | 2441 | 1161 | 2662 | 6,264 |

Table S5 Percentage of metagenomic reads/unique metabarcoding sequences passing FC1 corresponding to plant barcodes classified to species/genus/family

|  | Metagenomics | | | Metabarcoding | | |
| --- | --- | --- | --- | --- | --- | --- |
| Sample | Species | Genus | Family | Species | Genus | Family |
| BLM1 | 37.3 | 48.6 | 96.2 | 9.7 | 19.4 | 64.5 |
| BLM2 | 34.8 | 47.3 | 91.4 | 15.0 | 27.5 | 62.5 |
| BLM3 | 28.5 | 56.5 | 89.4 | 6.5 | 25.8 | 64.5 |
| BLM4 | 39.4 | 45.0 | 96.0 | 15.8 | 21.1 | 73.7 |
| BLM5 | 27.0 | 52.8 | 87.0 | 4.9 | 13.1 | 60.7 |
| BLM6 | 39.5 | 48.5 | 92.1 | 15.2 | 23.9 | 69.6 |

Table S6: SNP calling for MiSeq and HiSeq data using FreeBayes , ploidy=1

| Site (Annotation) | BLM1  Hiseq/Miseq | | BLM2  Hiseq/Miseq | | BLM3  Hiseq/Miseq | | BLM4  Hiseq/Miseq | | BLM5  Hiseq/Miseq | | BLM6  Hiseq/Miseq | |
| --- | --- | --- | --- | --- | --- | --- | --- | --- | --- | --- | --- | --- |
| 7791 (atp8) | 8/6 | 2/2 | 0/19 | 0/10 | 7/15 | 10/7 | 1/34 | 1/38 | 56/24 | 23/15 | 16/1 | 8/2 |
| 8155 (atp6) | 21/0 | 8/0 | 0/25 | 0/9 | 4/0 | 13/0 | 22/0 | 28/0 | 80/2 | 41/0 | 2/0 | 13/0 |
| 15572 (*d-*loop) | 6/32 | 8/9 | 0/20 | 0/10 | 2/32 | 4/14 | 6/34 | 2/31 | 57/70 | 31/30 | 15/1 | 14/3 |

Table S7: Identifications made by COI at 95% identity. All families with singleton identifications were excluded and genus/species level matches are shown if the meet they meet 98% identity criterion

| Order | Family | Genus | BLM1 | BLM2 | BLM3 | BLM4 | BLM5 | BLM6 |
| --- | --- | --- | --- | --- | --- | --- | --- | --- |
| Diptera | Muscidae |  | 2/-/- |  | 94/8/- |  |  | 16/2/- |
| Diptera | Sacrophagidae |  | 2/-/- |  | 94/2/- |  |  |  |
| Diptera | Drosophilidae | *Phortica* | 2/-/- |  | 94/2/1 |  |  |  |
| Diptera | Sepsidae | *Dicranosepsis* | 2/-/- |  |  |  |  | 16/3/2 |
| Lepidoptera |  |  |  |  |  |  |  | 5/-/- |

Table S8: Number of genera identified using single barcode. Correct: In Nee Soon checklist, Incorrect: Absent from Nee Soon checklist

| Sample | matK | | rbcL | | trnL-F | |
| --- | --- | --- | --- | --- | --- | --- |
|  | Correct | Incorrect | Correct | Incorrect | Correct | Incorrect |
| BLM1 | 19 | 4 | 15 | 4 | 25 | 15 |
| BLM2 | 27 | 8 | 12 | 1 | 25 | 16 |
| BLM3 | 17 | 3 | 10 | 4 | 15 | 14 |
| BLM4 | 13 | 2 | 11 | 3 | 17 | 6 |
| BLM5 | 20 | 5 | 14 | 3 | 26 | 14 |
| BLM6 | 35 | 12 | 23 | 8 | 42 | 17 |

References

1. Doyle J.J., Doyle J.L. 1987 A rapid DNA isolation procedure for small quantities of fresh leaf tissue. *Phytochemical Bulletin* **19**, 11-15.

2. Soltis D.E., Soltis P.S., Collier T.G., Edgerton M.L. 1991 Chloroplast DNA variation within and among genera of the Heuchera group (Saxifragaceae): evidence for chloroplast transfer and paraphyly. *American Journal of Botany* **78**(8), 1091-1112.

3. Kutty S.N., Pape T., Wiegmann B.M., Meier R. 2010 Molecular phylogeny of the Calyptratae (Diptera: Cyclorrhapha) with an emphasis on the superfamily Oestroidea and the position of Mystacinobiidae and McAlpine's fly. *Systematic Entamology* **35**(4), 614-635.

4. Kress, W.J., Erickson, D.L. 2007 A two-locus global DNA barcode for land plants: The coding rbcL gene complements the non-coding trnH-psbA spacer region. *PloS One* **2** (6), e508.

5. Taberlet P., Gielly L., Pautou G., Bouvet J. 1991 Universal primers for amplification of three non-coding regions of chloroplast DNA *Plant Molecular Biology,* **17,** 1105-1109.

6. Hunt T., Bergsten J., Levkanicova Z.,Papadopoulou A., John O.S., Wild R., Hammond P.M., Ahrens D., Balke M., Caterino M.S., et al. 2007 A comprehensive phylogeny of beetles reveals the evolutionary origins of a superradiation *Science*, **318**, 1913-1916.

7. Katoh K., Standley D.M. 2014 MAFFT: iterative refinement and additional methods. *Methods in molecular biology* **1079**, 131-146. (doi:10.1007/978-1-62703-646-7_8).

8. Ang A., Srivasthan A., Md-Zain B.M., Ismail M.R.B., Meier R. 2012 Low genetic variability in the recovering urban banded leaf monkey population of Singapore. *Raffles Bulletin of Zoology* 60(2), 589-594.
